# Diabetic Retinopathy Screening: A Systematic Review on Patients’ Non-Attendance

**DOI:** 10.3390/ijerph15010157

**Published:** 2018-01-19

**Authors:** Rahima Muhammad Kashim, Paul Newton, Omorogieva Ojo

**Affiliations:** Department of Adult Nursing and Paramedic Sciences, Faculty of Education and Health, University of Greenwich, Avery Hill Campus, Southwood Site, London SE9 2UG, UK; p.d.newton@gre.ac.uk (P.N.); o.ojo@gre.ac.uk (O.O.)

**Keywords:** diabetic retinopathy, retinal screening, patients’ non-attendance, systematic review

## Abstract

Diabetic Retinopathy is a microvascular complication of diabetes, that can go undetected and unnoticed until irreversible damage and even blindness has occurred. Effective screening for diabetic retinopathy has been proven to reduce the risk of sight loss. The National Health Service (NHS) which provides healthcare for all UK citizens, implemented systematic retinal screening for diabetic retinopathy in England in 2003, with the aim of identifying and treating all patients with sight threatening retinopathy. Crucial to this is patients partaking in the programme. Therefore, increasing screening uptake has been a major focus of the programme. This review explores the views of people living with diabetes who do not attend retinal screening, their characteristics, concerns, experiences of retinal screening and their understanding of the risks of diabetic retinopathy. All studies that satisfied the study inclusion criteria on ‘patients’ non-attendance at retinal screening’, between 2003 to 2017 were included after extensive database search. A total of 16 studies were included in the review. Findings showed that socio-economic deprivation was a major risk factor for non-attendance, about 11.5–13.4% of the screened population had sight threatening retinopathy (STDR), repeated nonattendance was linked to sight threatening diabetic retinopathy, and that certain factors, could be barriers or incentives for screening uptake. Some of those factors are modifiable whilst others are not.

## 1. Introduction

Diabetic retinopathy (DR) is a sight threatening, microvascular complication of diabetes that affects the retina. It is the most common complication of diabetes [1] and a leading cause of blindness amongst working aged adults in the developed world [2,3,4]. All persons with diabetes are at risk of developing retinopathy, however, persons living with type 1 diabetes (T1DM) have a higher chance of getting DR as compared to persons living with type 2 diabetes (T2DM) [5]. The prevalence of DR is directly linked to that of diabetes. Some studies estimate a DR prevalence of 34.6% [6,7] and find that it is more common in T1DM as compared to T2DM [7]. The exact mechanism of how prolonged hyperglycaemia causes retinopathy is still unclear, however studies have shown that prolonged hyperglycaemia alters retinal perfusion thereby disturbing the normal physiological and homeostatic state of the retina, in turn causing retinopathy [8]. Based on the presence or absence of abnormal blood vessels on the retina, it can broadly be classified into:

- Non-proliferative (NPDR)
- Proliferative (PDR) [9]

These can be further sub-classified into mild, moderate and severe retinopathy. Each level of classification has a different prognosis of vision with worst visual outcome associated with severe proliferative retinopathy. Maculopathy, which occurs when there are changes with the macula, functionally and severely affects vision, but may or may not be present with non-proliferative or proliferative retinopathy. In recent times, the natural history and factors that influence the development of DR have been understood a little better due to large trials and landmark studies in the UK and around Europe, making the management of DR better [10].

After the St. Vincent’s declaration to reduce blindness from diabetes by a third, the United Kingdom (UK) became the first country in the world to offer systematically organised screening for DR to all patients diagnosed with diabetes over the age of 12. This screening programme was implemented in England in 2003 and it reached nationwide coverage in 2008 [11]. The screening programmes within the four UK nations are overseen by a national programme and run at the community level by local programmes [12]. In England, screening is overseen by the National Diabetic Eye Screening Programme (NDESP) [13], in Wales it is the Diabetic Retinopathy Screening Service for Wales (DRSSW) [14], in Scotland, screening is run by the Scottish Diabetic Retinopathy Screening (DRS) [15] and in Northern Ireland, it is the Northern Ireland Diabetic Eye Screening Programme (DESP) [16]. Each of the four nations in the UK have some variations in their screening protocol and grading, but in general, retinal photographs are taken through a dilated pupil using non-mydriatic fundus camera. The photographs are then graded by specialist graders and the results/outcomes of the screening process are sent to hospital eye services if necessary for treatment, or to the patient’s general practitioner (GP), if no referable retinopathy is present. So, depending on the results, patients are either recalled for annual screening, invited back for more frequent surveillance or referred on to hospital eye services [11].

Apart from coordinating screening, the national programmes are also tasked with training, accreditation and quality assurance of the local programmes. In addition, they ensure the services go on smoothly and find ways to increase and improve the screening uptake. In England for instance, annual reports are put together showing number of patients invited, number screened, uptake, screening outcomes, referrals etc. within different regions. The 2016/2017 Public Health England’s report on uptake of retinal screening, showed that some areas neither met the acceptable uptake which is set at 70% by the National screening committee nor the optimal uptake rate (80%) [17].

To find out why some patients do not engage with these programmes despite its well-known and documented benefits, this review aims to bring together all existing studies on attendance, so that reasons for non-attendance can be explored.

## 2. Methods

To effectively answer the research question, a systematic review of all published and un-published literature on non-attendance at retinal screening was carried out, using the Preferred Reporting Items for Systematic Reviews and Meta-Analysis (PRISMA) checklist and flow chart for collecting and reporting data [18]. All studies on patients with type 1 diabetes or type 2 diabetes published after 2003 were included in the review. Prior to 2003 there was no systematic nationwide screening for retinopathy worldwide. In 2005, European countries made added commitments to the initial declaration in 1989, to have formal screening for [19]. Therefore, 2003 was chosen as the cut-off for including studies in this review, so that results from this review will not be affected by low screening uptake due to lack of an organised screening programme. In addition, all studies on healthcare providers’ perceptions of screening non-attendance were included, so as to have a better understanding of the reasons for patients’ non-attendance and also to not severely restrict the scope of the study. There were no restrictions placed on patients’ characteristics such as age, sex, duration of diabetes, location, ethnicity, or country of origin. Excluded in this review were: studies carried out before 2003 even if they were published after 2003, studies that were written in any language other than English, clinical audits, and studies that sought to look at interventions to increase screening uptake.

A scoping search using Google Scholar was carried out to build up appropriate search terms to be used for identifying articles for the study. After which, Wiley Online, Web of science, Ebscohost, and Science Direct were searched for articles related to patients’ non-attendance and retinal screening. The following keywords were used: ‘Patients’, ‘non-attendance’ ‘Retinal Screening’ and ‘Barriers to access’ (see details of the search terms used and their combinations in Appendix A Table A1 and Table A2).

**Figure 1:**
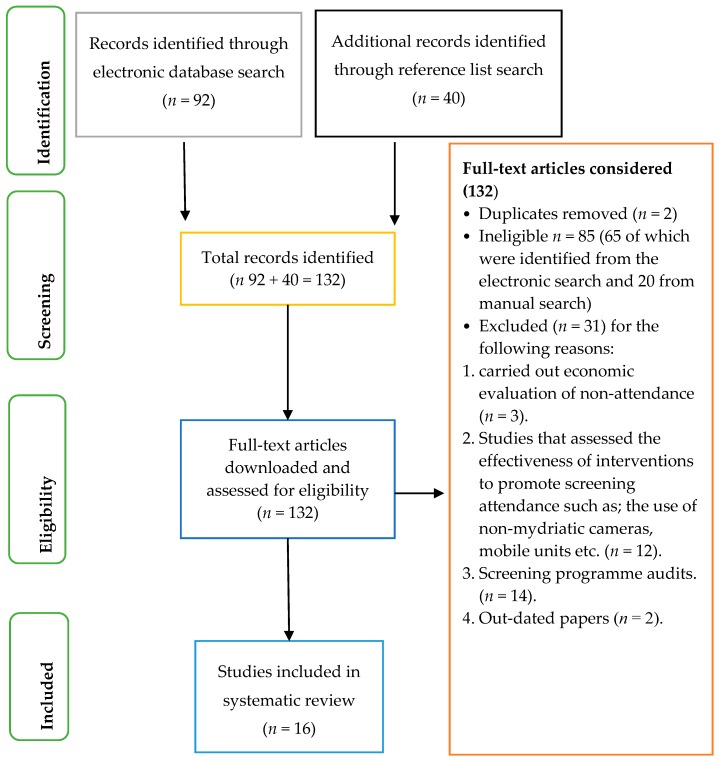
Flow chart showing the article selection process.

Identified papers for inclusion were downloaded and assessed using the McMaster critical appraisal tool [20] and were scored out of 15 for quantitative studies and out of 16 for qualitative studies. A score of 10 was used as the cut off mark for the studies, and only studies with 10 or above were used in the review. A simple table in Excel was designed to extract the same data from all included studies, such as; study country, participants, study design, methods, main aim and outcome etc. A general inductive approach to data analysis was done, to allow for thematic analysis of the data through rigorous and repeated studying of extracted data and transcripts of the included studies, grouping segments of texts by themes was done until no new theme emerged from the study. Below is a flow chart showing the study selection process (see Figure 1).

## 3. Results

A total of 132 articles were downloaded, 16 of which were included in the review after critically appraising the articles. Eleven of the included studies were carried out in the United Kingdom and one each in Ireland, Iceland, The Netherlands, Saudi Arabia and United States of America. Eight of the included studies were quantitative studies and 8 qualitative studies. The sample size within each study varied largely from below 100 to tens of thousands in other studies. The table below summarises the characteristics of the included studies, their main aims and outcomes.

Five main themes emerged from critically studying the data and the results of the included studies. The themes were: (1) Demographics of non-attending patients (2) screening invitations; screening uptake and screening outcome (3) facilitators and barriers to screening compliance (4) patients’ perceptions and their screening experiences (5) factors that could contribute to better screening uptake. Below are details of the identified themes (see Table 1).

### 3.1. Demographics of Non-Attenders

**Figure A1:**
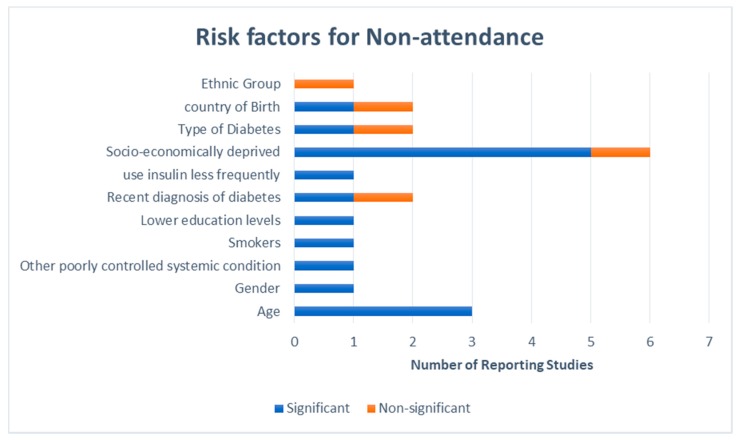
Chart showing risk factors within a population that influences their screening uptake.

Two of the included studies, [28,31] identified characteristics such as living in socially deprived areas, being young (age), having poor glycaemic and blood pressure control, smoking, having lower education, a more recent diagnosis of diabetes and less frequent use of insulin shaped non-attendance at retinal screening programmes. Socio-economic deprivation was the most referenced demographic characteristic of non-attenders, with five studies [4,29,30,31,32,33] reporting that non-attendance increased with higher socio-economic deprivation and that there was a significant and large difference between the least and most deprived quintile of deprivation in terms of screening attendance using the indices of English deprivation (IED). The second most referenced characteristic of non-attenders was age, all the studies that made mention of the age of non-attenders found that non-attendance was highest amongst younger patients [30,31,32,33], and then the much older patients [30], then ethnic minorities, and being born outside the UK or Republic of Ireland (see Appendix A Figure A1).

### 3.2. Screening Invitation, Uptake and Outcome

**Figure A2:**
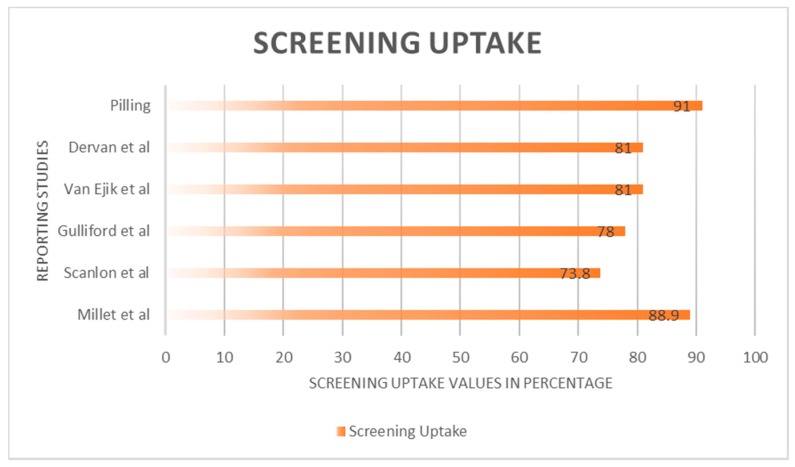
Reported screening uptake.

Screening uptake ranged from 61–88.9% amongst the diverse study participants in the individual studies, however not all eligible patients were invited for screening with only 46% of eligible patients reported to have been invited in South East London [33]. The percentage of sight threatening retinopathy identified after screening ranged from 11.5% [30]–60% [34], and that poor visual outcome, and an increased risk of sight threatening retinopathy were associated with poor compliance and repeated non-attendance respectively [27,34] (see Appendix A Figure A2).

### 3.3. Facilitators and Barriers to Screening Compliance

Facilitators for screening were identified as:

- A recommendation from a healthcare provider [21,25,28,32] and,
- Knowledge about effects of non-attendance on vision [21,25,28,32].

Patient level barriers to screening included:

- Having competing priorities [23],
- Anxiety about the screening [23],
- Disengagement with diabetes care [23],
- Misinformation about screening [23] and,
- Forgetting to attend for the screening [23].

System level reasons for patients’ non-attendance were:

- issues about patients’ addresses [23],
- not sending the screening invitations out on time [23] and,
- patients’ clinical notes not being shared [23].

### 3.4. Patients’ Perceptions and Experiences of Screening

Misunderstanding the reason for screening and the screening process were noted by a number of studies [22,25,32,35] Patients gave negative accounts of their experiences of screening and the screening process. The most cited of which were; lengthy appointment/waiting times, pain discomfort and other side effects of the drops used to dilate the pupils before the screening and lastly, not being able to be independent for some time after their pupils have been dilated [25,32].

### 3.5. Factors that Could Contribute to Better Screening Uptake

Factors such as improving communication between GP practices and screening services, improving ways in which patients are invited and contacted for the screening, integrating retinal screening with other diabetic care, properly educating, inducting and integrating newly diagnosed diabetic patients into the screening programme, changing the way staff behave towards previous non-attenders, trying to bridge the language barrier gap, and making the programmes sensitive to differences of ethnic minorities, and placing screening sites in areas that are easily accessible and have good transport links [26] can be modified in order to increase screening uptake.

## 4. Discussion

This review has provided an in-depth understanding on patients’ non-attendance at retinal screening programmes. The mixture of quantitative and qualitative studies in this review plays a complementary role in not only identifying the reasons for non-attendance but also explaining them from not only the healthcare professionals’ views but patients’ also. It is very important to note that the screening programmes must fully understand their patients, their sensitivities and challenges and must cater to the needs of the population if the desired outcome is meant to be achieved. For instance, in the study by Leese et al., in Tayside, Scotland, a sub-urban largely white population, they not only noted the characteristics of non-attenders, but interestingly they found that travel distance did not affect attendance, and that attendance was considerably better amongst patients that attended the static screening services as compared to mobile screening services [31]. This is contrary to findings from Lindenmeyer et al. that found that transportation and access to the screening site was a factor that determined screening uptake [26]. This finding could mean that when patients know exactly where their screening venue is, they are able to plan and make the required commitments to attend the screening as compared to a screening venue that changes. It could also be that patients did not look forward to being screened in a van. A qualitative study will be required to fully understand why this was found.

Secondly, even though there was a relationship between socio-economic deprivation and screening non-attendance in all the studies that reported the finding, the level of difference identified varied within the different studies [4,29,30,33]. In Scanlon et al. study, they noted a difference of 9.3% between the least and most deprived quintiles [4]. In the Millet et al. study however, the difference was much less with only a 3.8%, between the least and the most deprived quintiles [33]. However, unlike in the Scanlon et al. study where they considered county wide data for its comparison of uptake and socio-economic deprivation, Millet et al. used data from only a few South-East London boroughs, which form some of the most deprived places in England; this could be the reason for the much lower difference noted in their study because of less disparity in their data to begin with. The study by Waqar et al. [29], found a difference of 9.4% which is similar to the 9.3% found by Scanlon et al. [4]. However, when they further classified the ‘do not attend’ (DNAs) patients into first time DNAs (DNA1) and Repeat DNAs (DNA2), the deprivation analysis showed that there was a difference of 4.9% between the most and least deprived quintiles among first time non-attenders and a 2.2% difference amongst repeat non-attenders. These findings are similar to that of Millet et al. [33] and Guilford et al. [30].

Results of the screening uptake were mostly above the optimal level of uptake set by the NDESP, however generalising the results of screening uptake or inferring that screening uptake is good would be problematic because the study locations and study population were very different, and the studies had individual cut-offs for what non-attendance meant. For instance, in the study by Van Ejik et al. [28], screening uptake was 81%, however non-attendance was defined as not having attended in the past three years, and accounts of attendance were self-reported through questionnaires that had a 73% response rate. Whereas in the study by Strutton et al. [23], they defined non-attendance as not having attended screening in the last 18months. The study by Pilling [26], reported a 65% screening uptake which was in a very specific population of patients with diabetes and learning disabilities. Ninety-one percent of the study’s population were offered screening. Which is very different to findings by Millett and Dodhia [33] where only 46% of the eligible population were invited for screening with 88.9% of them partaking in the screening programme.

It is clear that the importance of screening cannot be overemphasized. In the Zoega et al. study, they compared a group of registered blind patients on the Icelandic register to age matched controls and found that patients registered as blind had a significantly lower level of pre-diagnosis screening compliance [34]. Similarly, Forster et al. found that patients who were not screened for two years before re-attending screening had 10.84 times higher odds of referable retinopathy being detected when compared to participants who were screened each year [27]. There was no risk of referable retinopathy for participants who did not attend for screening in one year. They observed the same pattern for referable maculopathy and STDR though the effect sizes were smaller [27].

Despite the known effects of non-attendance at screening, some patients still do not engage with these programmes. When looking into barriers and facilitators to screening, most of the studies had similar findings as mentioned above, except for the study by Hartnett et al. [35] and Al-Alawi et al. [22] which had unique barriers to screening. Hartnett et al. [35] cited finances as a major barrier to screening; it is easy to see why that would be a barrier within their study population, because in the US, healthcare must be paid for at the point of access unlike the UK and most of Europe. The study by Al-Alawi et al. [22] in Saudi Arabia, also found a barrier to screening that had not been mentioned by other studies which was a lack of gender specific screening professionals. This finding is an interesting one as it shows how health seeking behaviour can be influenced by religious and cultural beliefs. This finding needs to be explored in depth to find ways of improving screening uptake within the concerned population.

### Strengths and Limitations

The rigour and explicit methods of searching for studies, critically appraising the included studies in order to reduce reporting on studies with a flawed methodology, and thematic analysis to preserve findings from the primary studies and allow for a more transparent link and comparison are some of the strengths of this study.

An outcome bias might have been introduced, because non-attendance at screening programmes was the outcome of choice for this study, therefore only studies reporting on non-attendance were included in the review and that is a limitation of the study.

## 5. Conclusions

The current evidence clearly suggests that screening uptake is less than optimal, and identifies characteristics of non-attendance and reasons for non-attendance. However, there are some limitations in interpreting the evidence provided, first because there is not a clear definition of non-attendance, and secondly largely categorising patients into ‘ethnic minority’ or ‘socio-economically deprived’ does little to help our understanding of why the different segments of people within those large classifications do not attend for retinal screening, e.g., young people. More qualitative research looking in-depth at which patients do not attend, and seeking to understand why they do not attend, and how the screening programme can be modified to get them to attend, is needed.

## Figures and Tables

**Table 1:** Showing characteristics of included studies.

Studies	Participants	*N*	Methods Used	Aim	Main Outcome
Lake et al. 2017 (UK) [21]	Young adults with T2D	30	Structured interviews	Explore factors associated with non-attendance	Some factors such as social influences, etc. are peculiar to younger adults with T2D.
Al-Alawi et al. 2016 (Saudi Arabia) [22]	Hospital staff with diabetes	45	close ended questionnaire	Evaluate knowledge, attitudes and barriers to screening	Most of the participants had excellent knowledge about diabetic eye complications, absence of gender specific-professionals was a cause for non-attendance.
Strutton et al. 2016 (UK)[23]	Screening DNAs (≥18 months)	146	Telephone Interview	Identify patient and system level reasons for non-attendance	Patients reasons included, having other commitments, anxiety etc. and system level reasons were mostly about miscommunication.
Piling et al. 2015 (UK) [24]	Diabetics with learning disability	71	Retrospective analysis	Find out if there is equality of access for patients with learning disability	National standards are not met for diabetic patients with learning disabilities
Hipwell et al. 2014 (UK) [25]	Staff and diabetic patients	62	Semi-structured interviews	Examine experiences with screening and how that affects uptake	Knowledge of DR was a major antecedent to screening, and psychological, pragmatic and social factors were antecedents to non-attendance.
Lindenmeyer et al. 2014 (UK) [26]	GPs	9 GP practices	Semi-structured interviews	Identify factors that contribute to screening uptake	Some factors are modifiable such as improving communication, others were not such as diversity of ethnicity and languages.
Forster et al. 2013 (UK) [27]	Patients first screened in 2008	6556	Retrospective analysis	Evaluate whether repeated non-attendance was linked to STDR	Repeated non-attendance increases risk of STDR.
Van Eijk et al. 2012 (The Netherlands) [28]	Diabetic patients ≥ 18 years	2363	Focus groups & Questionnaires	Examine barriers and incentives to screening	81% attendance; non-attenders had lower education levels, shorter duration of diabetes and were less likely to use insulin or be checked by and internist.
Waqar et al. 2012 (UK) [29]	Screening DNAs (04/09–03/10)	22,651	Retrospective analysis	Evaluate relationships between socio-economic status and non-attendance	Increasing non-attendance with deprivation, lowest DNAs seen in successful professionals and highest DNAs seen in areas of social housing
Gulliford et al. 2010 (UK) [30]	Diabetic patients on a screening database	59,495	Retrospective analysis	Quantify socio-economic and ethnic in-equalities in screening	Only a weak association between non-attendance and deprivation, and smaller than previously reported inequality in screening.
Leese et al. 2008 (UK) [31]	Diabetic patients on a screening database	15,150	Retrospective analysis	Identify characteristics that determine attendance status	Socio-economic deprivation increases non-attendance
Dervan et al. 2008 (Ireland) [32]	Patients at diabetic centres	209	Questionnaires	Assess if patients are receiving regular screening and factors that influence uptake.	81% of patients had screening done. Recommendation from a physician was a major factor in increasing uptake.
Scanlon et al. 2008 (UK) [4]	Diabetic patients on a screening database	10,312	Retrospective analysis	Investigate socio-economic differences in prevalence of diabetes, DR and screening uptake	Probability of being screened decreased with deprivation, prevalence of diabetes and developing STDR was associated with increasing deprivation
Millet et al. 2006 (UK) [33]	Diabetic patients on a screening database	9750	Retrospective analysis	Assess screening and screening outcome in South East London	88.9% screening uptake, however, significant inequity in the delivery of the screening programme.
Zoega et al. 2005 (Iceland) [34]	Patients on the Icelandic blind registry	22	Retrospective analysis	Find relationship between screening compliance and visual outcome	Blind patients had worse pre-diagnosis screening compliance
Hartnett et al. 2005 (USA) [35]	Diabetic patients and physicians at an indigent clinic	2145 records, 17 focus group participants	Focus group discussions and interviews	Address inadequate screening and explore perceived barriers.	Physicians and patients have different perceived barriers to screening, though they both agreed that accessing the care was also a barrier

## Appendix A

**Table A1:** Search terms.

Index Term	Synonyms	Related Terms
Patients non-attendance	(1a) Patients no show (1b) Patients absenteeism (1c) do not attend (1d) non-compliance (1e) non-concordance	(1f) AWOL (1g) non- appearance (1h) Barriers to access
Retinal Screening	(2a) Diabetic eye test (2b) Diabetic screening (2c) Diabetic Retinopathy	(2d) retinal monitoring (2e) Retinal photography (2f) Retinal checks (2g) Diabetic maculopathy
motives	(3a) Attitudes (3b) Reason (3c) Rationale (3d) Experiences	(3e) Drive (3f) Stimulus (3g) Basis

**Table A2:** Search strategy.

Date	Search Terms Used	No. of Papers Excluded	No. of Papers Included
Ebscohost			
20/3/17	1 [OR] 1a and 2	10	2
	1 [OR] 1c and 2	30	7
22/3/17			
	1c and 2a	2	0
	1 and 2b	3	2
	1 and 2e	1	0
	1c and 2 and 3d	2	1
Web of Science			
24/3/17	1 [OR] 1a and 2	1	2
	3 [OR] 3d and 2	95	7
Science Direct			
27/3/17	3d and 2	2018	6
	1c and 2	1908	4
	1 and 2 [OR] 2b	124	0
	1a [OR] 1c and 2	39,708	2
	1a [OR] 1h and 2	2686	9
	1a–1h and 2 [OR] 2b		8
Cochrance Library			
03/4/17	3d and 2	24	0
	3d and 2		1
Nice Database			
06/4/17	NICE and 2d	215	35
Scopus			
15/06/17	1 [OR] 1c and 2	5	1
	3 [OR] 3d and 2 [OR] 2b and 1	7	5
			Total: 92
